# Regulation of Host Translational Machinery by African Swine Fever Virus

**DOI:** 10.1371/journal.ppat.1000562

**Published:** 2009-08-28

**Authors:** Alfredo Castelló, Ana Quintas, Elena G. Sánchez, Prado Sabina, Marisa Nogal, Luis Carrasco, Yolanda Revilla

**Affiliations:** Centro de Biología Molecular Severo Ochoa, CSIC-UAM, Universidad Autónoma de Madrid, Madrid, Spain; New York University, United States of America

## Abstract

African swine fever virus (ASFV), like other complex DNA viruses, deploys a variety of strategies to evade the host's defence systems, such as inflammatory and immune responses and cell death. Here, we analyse the modifications in the translational machinery induced by ASFV. During ASFV infection, eIF4G and eIF4E are phosphorylated (Ser1108 and Ser209, respectively), whereas 4E-BP1 is hyperphosphorylated at early times post infection and hypophosphorylated after 18 h. Indeed, a potent increase in eIF4F assembly is observed in ASFV-infected cells, which is prevented by rapamycin treatment. Phosphorylation of eIF4E, eIF4GI and 4E-BP1 is important to enhance viral protein production, but is not essential for ASFV infection as observed in rapamycin- or CGP57380-treated cells. Nevertheless, eIF4F components are indispensable for ASFV protein synthesis and virus spread, since eIF4E or eIF4G depletion in COS-7 or Vero cells strongly prevents accumulation of viral proteins and decreases virus titre. In addition, eIF4F is not only activated but also redistributed within the viral factories at early times of infection, while eIF4G and eIF4E are surrounding these areas at late times. In fact, other components of translational machinery such as eIF2α, eIF3b, eIF4E, eEF2 and ribosomal P protein are enriched in areas surrounding ASFV factories. Notably, the mitochondrial network is polarized in ASFV-infected cells co-localizing with ribosomes. Thus, translation and ATP synthesis seem to be coupled and compartmentalized at the periphery of viral factories. At later times after ASFV infection, polyadenylated mRNAs disappear from the cytoplasm of Vero cells, except within the viral factories. The distribution of these pools of mRNAs is similar to the localization of viral late mRNAs. Therefore, degradation of cellular polyadenylated mRNAs and recruitment of the translation machinery to viral factories may contribute to the inhibition of host protein synthesis, facilitating ASFV protein production in infected cells.

## Author Summary

African Swine Fever Virus (ASFV) is a large DNA virus that infects different species of swine, causing an acute, highly contagious and often fatal disease. Infection by ASFV is characterized by the absence of a neutralizing immune response, which has so far hampered the development of a conventional vaccine. While a number of reports have been concerned with ASFV genes and mechanisms regulating programmed cell death and immune evasion, nothing is known so far regarding how ASFV replicates in the infected cells. As intracellular parasites, viruses are highly dependent on host translation machinery for synthesizing their own proteins. We have observed that the cellular protein synthesis is strongly inhibited during ASFV infection, while viral proteins are efficiently produced. Furthermore, we here describe the processes by which ASFV activates and redistributes the cellular machinery to synthesize its own proteins. It has been reported that ASFV replicates within discrete cytoplasmic areas known as factories. In this regard, we have identified the presence of important cellular factors involved in the control of protein synthesis, located close to viral factories, together with ribosomes and the mitochondrial network, which represents a sophisticated mechanism of viral control.

## Introduction

**Figure 1:**
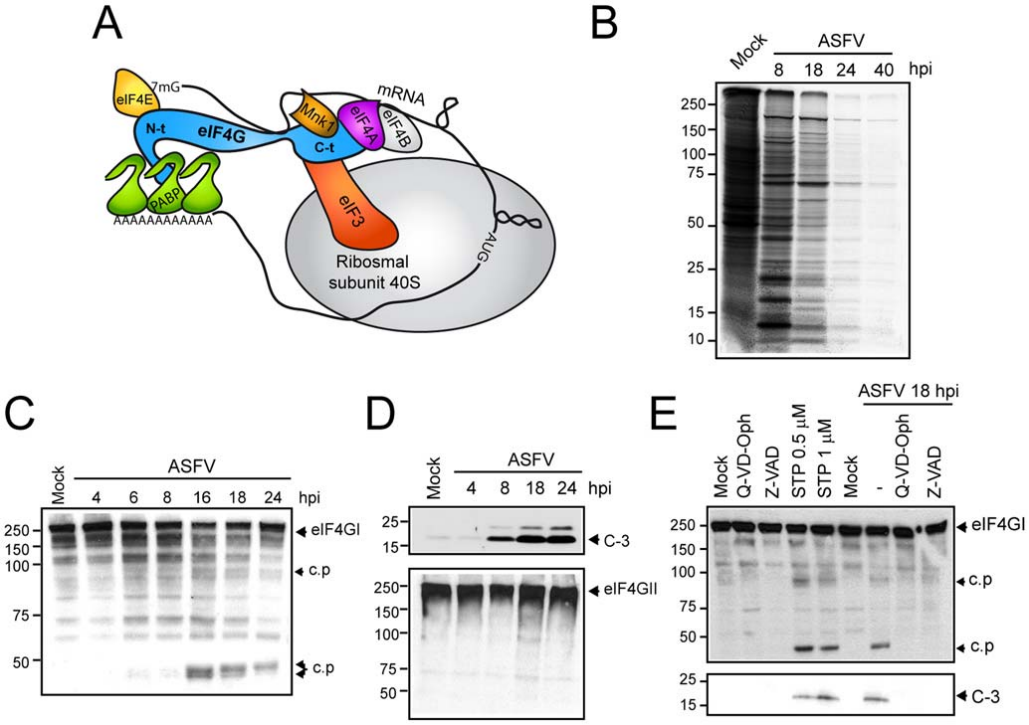
Inhibition of host protein synthesis and analysis of caspase-3-mediated eIF4GI cleavage in ASFV-infected cells. A) Schematic representation of translation initiation complex. B) Cellular and viral protein synthesis during ASFV infection. Cultures of Vero cells (5×10^5^) were mock infected (Mock) or infected with ASFV (5 pfu/cell), and labeled at different times after infection with 200 µCi of [^35^S]Met-[^35^S]Cys/ml in cysteine-methionine-free medium for 2 h. Samples were analyzed by SDS-PAGE followed by fluorography and autoradiography. C) Activation of caspase-3 during ASFV infection induces incomplete eIF4GI degradation. eIF4GI was detected by Western blot by incubation with specific antiserum at the indicated times after ASFV-infected Vero cells. D) eIF4GII (upper panel) and cleaved caspase-3 (bottom panel) were analyzed by Western blotting. E) Analysis of eIF4G1 cleavage by using specific caspase-3 inducers or inhibitors in mock-infected or ASFV-infected cells. Vero cells were treated with 0.5 or 1 µM staurosporin or were infected with ASFV (5 pfu/cell). Two replicates of mock-infected or ASFV-infected cells were treated with 60 µM Q-VD-Oph or Z-VAD. Cells were recovered after 18 h in sample buffer and eIF4GI (upper panel) and caspase-3 (bottom panel) activation was analyzed by Western blot. c.p., cleavage product; C-3, cleaved caspase-3 (17 KDa); STP, staurosporin.

The vast majority of animal cytolytic viruses interfere with cellular gene expression after infection of host cells. Cellular protein synthesis in particular is usually abrogated at times when late viral proteins are being synthesized [1]–[3]. However, the molecular mechanisms by which viruses induce this phenomenon are still under investigation. Eukaryotic initiation factor (eIF) 4F is composed of eIF4E, eIF4A and eIF4G (Figure 1A). eIF4E binds the cap structure present at the 5′ end of cellular mRNAs; eIF4A is an RNA helicase that unwinds the secondary structure near to the initiation codon and eIF4G is a scaffolding protein that physically links the mRNA and the small ribosomal subunit by means of several protein-protein interactions [4]. In particular, the N-terminal domain of eIF4G binds to eIF4E and poly(A)-binding protein (PABP), which are involved in mRNA recruitment through interaction with the cap and the poly(A) tail, respectively (scheme in Figure 1A) [5],[6]. The C-terminal domain of eIF4G interacts with eIF4A, mitogen-activated kinase 1 (Mnk-1) and eIF3, a complex eIF that binds the 40S ribosomal subunit [4]. In addition, eIF4F functions are regulated by extra- and intracellular signals in eukaryotic cells by means of phosphorylation processes [7],[8]. In example, Pak-2 phosphorylate eIF4GI at the eIF4E-binding site, inhibiting the interaction between both factors [7]. On the other hand, eIF4E is phosphorylated by the protein kinase Mnk-1, although the role of this event is still under investigation [2],[9]. In addition, there are several eIF4E-binding proteins (4E-BPs) that have the ability to sequester eIF4E in a phosphorylation-dependent manner [9],[10]. In their hypophosphorylated form, 4E-BPs inhibit cap-dependent translation on interacting with eIF4E. In contrast, hyperphosphorylation of 4E-BPs by mTOR kinase leads to their release from eIF4E and subsequent association with eIF4G, thereby assembling an active eIF4F complex [9],[10].

Given the essential role of eIF4F in cellular mRNA translation, it is not surprising that many animal viruses target eIF4F during the viral cycle [2],[3]. This is the case of some picornaviruses, retroviruses and caliciviruses, which encode proteases that cleave eIF4G, separating its N-terminal and C-terminal domains [11]. Other viruses such as encephalomyocarditis virus (EMCV), adenoviruses (AdV) or vesicular stomatitis virus (VSV) induce the dephosphorylation of eIF4E and 4E-BPs, leading to inactivation of cap-dependent translation [12]–[15]. By contrast, mRNAs synthesized by some DNA viruses, such as herpes simplex virus type 1 (HSV-1), human citomegalovirus (HCMV) or vaccinia virus (VV), are translated by a cap-dependent mechanism. These viruses can in fact stimulate eIF4F assembly to enhance viral protein synthesis [16]–[19]. In addition to eIF4F, the phosphorylation of eIF2α may play a key role in the regulation of cellular and/or viral translation [3]. eIF2α is a subunit of eIF2, a multiprotein complex responsible for binding the initiator Met-tRNA_i_ to the 40S ribosomal subunit. Phosphorylation of eIF2α impairs the exchange of GDP for GTP mediated by eIF2B [20],[21]. In mammalian cells, four protein kinases are able to phosphorylate eIF2α, but the double-stranded RNA-activated kinase PKR is of major importance in the regulation of translation during viral infection [22]. Subsequently, some animal viruses have developed different strategies to counteract PKR activation [3].

African swine fever virus (ASFV), the sole member of the Asfarviridae family [23], is a large and complex cytoplasmic DNA virus of icosahedral symmetry that infects different species of swine, causing acute and often fatal disease. Infection by ASFV is characterized by the absence of a neutralizing immune response, which has so far hampered the development of a conventional vaccine. ASFV replicates exclusively within the host cell cytosol, although a nuclear step has been also reported [24]. Indeed, discrete cytoplasmic areas are reorganized into replication sites, known as factories, during the productive virus cycle [25]. Analysis of the complete DNA sequence of the 170-kb genome of the BA71V isolate, adapted to grow in Vero cells, has revealed the existence of 151 genes, including those coding for structural proteins, a number of enzymes with functions related to DNA replication, gene transcription and protein modifications, as well as several genes able to modulate virus-host interaction. Moreover, ASFV induces the activation of caspase-3 and p53 and is able to interfere with inducible gene transcription, leading to immune evasion [26]–[29]. Transcription of ASFV genes gives rise to cap and polyadenylated mRNAs, however, the regulation of translational machinery in ASFV-infected cells has not been studied. In spite of encoding a variety of enzymatic activities [30],[31], ASFV is fully dependent on the cellular translational machinery to synthesize viral proteins. In the present work, a number of eIFs are analyzed in ASFV-infected cells. We provide evidence that eIF4E and eIF4G are phosphorylated at Ser209 and Ser1108 respectively and these events strongly correlate with a robust viral protein synthesis and an increase of eIF4F assembly. Inhibition of either eIF4GI or eIF4E phosphorylation partially affects viral protein synthesis and virus spread, whereas the knock down of these factors strongly avoid ASFV infection. On the other hand, eIF4GI and eIF4E are recruited within ASFV factories at 8 hpi, and they are later redistributed to the periphery of these particular foci. Finally, eIF4GI, eIF4E, eIF2α, eIF3b, eEF2 and ribosomes are closely distributed to ASFV factories at 16 hpi, being ASFV late mRNAs and mitochondrial network found at these areas.

## Materials and Methods

### Cell culture, viruses, and reagents

Vero and COS-7 (African green monkey kidney) cells were obtained from the American Type Culture Collection (ATCC) and grown in Dulbecco's Modified Eagle's Medium supplemented with 5% fetal bovine serum (Invitrogen Life Technologies). Cells were grown at 37°C under a 7% CO_2_ atmosphere saturated with water vapor in a culture medium supplemented with 2 mM L-glutamine, 100 U/ml gentamicin and nonessential amino acids. The Vero-adapted ASFV strain Ba71V was propagated and titrated by plaque assay on Vero cells, as described [26]–[29]. Infection of Vero and COS-7 cells with Ba71V ASFV was carried out at a multiplicity of 1–5 pfu/cell. Silencing was achieved by transfecting COS-7 or Vero cells twice (0 and 24 h) with 25 nM siRNAs (siControl [Gene Link], si4E [TACATTAATCGGTAGCAGGAA] [32], si4GII-2 [CAAAGACCTGGACTTTGAA] (EW, AC, PM and LC, unpublished data) and si4GI-31 [CCCAUACUGGAAGUAGAAGTT] [33]) using lipofectamine 2000 (Invitrogen) according to the manufacturer's recommendations. Chemical inhibitors were dissolved as 1000× stocks in DMSO and used at the concentrations indicated. Rapamycin (Calbiochem) and CGP57380 (Sigma) were used at 250 nM and 20 µM, respectively. Cells were pretreated with rapamycin or CGP57380 12 h before infection with ASFV and the experiments were carried out in the continuous presence of either inhibitor. Apoptosis was induced using 0.5 or 1 µM staurosporin (Sigma). Caspase activity was prevented by incubation with 60 µM Q-VD-Oph (Calbiochem) or Z-VAD fmk (Bachem). Cytosine arabinoside (AraC) (Sigma) was used at 40 µg/ml.

### Protein synthesis and Western blot analysis

Synthesis of cellular and ASFV proteins was analyzed by metabolic labelling with 200 µCi of [^35^S]Met-[^35^S]Cys/ml (Promix; Amersham Biosciences) for 2 h, followed by SDS-PAGE, fluorography and autoradiography. Mock-infected or ASFV-infected cells were washed twice with PBS and lysed in lysis buffer or RIPA modified buffer supplemented with protease and phosphatase inhibitor cocktail tablets. The protein concentration was determined by the bicinchoninic acid spectrophotometric method (Pierce). Cell lysates (30 µg of protein) were fractionated by SDS-PAGE and electrophoretically transferred to an Immobilon extra membrane (Amersham). For autoradiography, the gel was exposed on a Fujifilm BAS-MP 20405 imaging plate at room temperature. The exposed imaging plate was analyzed with a Fuji BAS 1500 analyzer. The steady-state level and the integrity of initiation factors were analyzed by Western blot. eIF4GI was detected with antisera raised against peptides derived from the N-terminal and C-terminal region of human eIF4GI at 1∶1000 dilution [34]. Rabbit antisera against N-terminal and C-terminal region of eIF4GII (a generous gift from N. Sonenberg, McGill University, Montreal, Canada) were employed at 1∶500 dilution. eIF4A was detected with a mouse monoclonal antibody (a generous gift from Dr. H. Trachsel, Institute for Biochemistry and Molecular Biology, University of Berne, Switzerland) at 1∶50 dilution. Anti-eIF2α, anti-p-eIF4E (Ser209), anti-eEF2, anti-Mnk-1, anti-PKR (Santa Cruz Biotechnology), anti-p-eIF2α (Ser52) (Invitrogen), anti-eIF4E (BD Transduction laboratories), anti-p-eIF4GI (Ser1108), anti-Caspase-3, anti-p-Mnk-1 (Thr197/202) (Cell Signalling), anti-α-Actin, anti-PTB and anti-α-Tubulin (Sigma) were employed according to the manufacturer's recommendations. Anti-4E-BP1 (Santa Cruz Biotechnology), anti-nonphospho-4E-BP1 (Thr46) and anti-phopho-4E-BP1 (Thr70) (Cell Signalling) were used at 1∶1000 dilution. To detect ASFV proteins, polyclonal antibodies raised against p72 or p32 polypeptides or against complete ASFV virus, were used at 1∶1000 dilution. Anti-rabbit, anti-mouse (Promega) and anti-goat (Santa Cruz Biotechnology) immunoglobulin G antibodies coupled to peroxidase were used at 1∶5000 dilution. The percentage of protein synthesis and steady-state levels of each protein was estimated by densitometric scanning of the corresponding band.

### Sepharose-4B-m7GTP matrix

Vero cells were infected with 3 pfu/cell of ASFV. At the indicated times, cells were harvested and lysed in buffer A (150 mM NaCl, 1.5 mM MgCl_2_, 10 mM Tris-HCl [pH 8.5], 0.2% Igepal) with proteases, phosphatases, and RNase inhibitors as described above. The lysates were centrifuged at 10,000 × *g*, and the supernatants were incubated with Sepharose-m7GTP (Amersham) or Sepharose-4B (Sigma), as a negative control, overnight at 4°C. The resins were washed five times with buffer A, resuspended in Laemmli sample buffer, and analyzed by SDS-polyacrylamide gel electrophoresis and Western blotting [35].

### Immunofluorescence microscopy and FISH

Fixation, permeabilization and confocal microscopy were performed as described previously [36], employing a confocal LSM510 coupled to an Axiovert 200 M microscope (Zeiss). A rabbit antibody raised against the C-terminal domain of eIF4GI was used at 1∶1000 dilution. Monoclonal anti-eIF4E antibody was employed at 1∶100 dilution. eIF2α, eIF3b (p110) and eEF2 was detected with the corresponding antibodies at 1∶50 dilution. ASFV p72 protein was localized by using specific rabbit or mouse antibodies generated in our laboratory. Eukaryotic ribosomal P protein was detected with a mouse monoclonal antibody (a generous gift from J.P. García-Ballesta, Centro de Biología Molecular “Severo Ochoa”, Madrid) (1∶10 dilution) [37]. For mitochondria staining, cells were incubated with 2 µM MitoTracker red CMH2-Ros (Molecular Probes) for 45 min before fixation. Fluorescence *in situ* hybridization (FISH) with fluorescein labeled oligo d(T) (Gene Link) was carried out as previously reported [38]. FISH using specific fluorescein-tagged probes against A224L [GCTTTGATTTCGTGCATCTATGGAGC] and p72 [CGCAGGTGACCCACACCAACAATAACCAC] mRNAs was carried out as follows: cells were fixed and permeabilized and then washed tree times: first with PBS 1X, the second washed with PBS 1X and SSC 1X and the third one with SSC 2X. Next, cells were incubated at 37°C with the pre-hybridation buffer (SSC 2X, 20% deionized formamide, 0.2% BSA and 1 mg/ml yeast tRNA). After, cells were incubated at 42° with the hybridation buffer (SSC 2X, 20% deionized formamide, 0.2% BSA, 1 mg/ml yeast tRNA, 10% dextran sulphate and 1 pmol/µl of either p72 or A224L probes) for 4 h. Preparations were washed four times at 55° for 5 min: the first washed was performed with SSC 2X mixed with 20% formamide; the second one was carried out with SSC 2X; the third one with SSC 1X and PBS 1X and the last one with PBS 1X. FISH was followed with the immunofluorescence protocol. Image processing was performed with Huygens 3.0 software.

### Cell processing for electron microscopy

At the times indicated, Vero cells were fixed with 2% glutaraldehyde in 0.2 M HEPES buffer (pH 7.4) for 1 h at room temperature and immediately scraped off the plate. For immunoelectron microscopy, cells were processed by freeze substitution. Immunogold localization of eukaryotic ribosomal P protein was done by placing the ultrathin sections on drops of different solutions. After incubating for 30 min with TBG (TBS [Tris-HCl 30 mM, NaCl 150 mM, pH 8.2] supplemented with 0.1% BSA and 1% gelatin from cold water fish skin), sections were floated for 1 h on a drop of anti-P antibodies diluted in TBG. The grids were then washed in TBS supplemented with 0.1% BSA (3×5 min) and then exposed to 10 nm colloidal gold conjugated goat anti-rabbit IgG diluted in TBS for 1 h. Then, the grids were washed consecutively with TBG, TBS, and distilled water (5 min each) before being stained with a solution saturated of uranyl acetate followed by lead citrate.

### Accession numbers

#### ASFV proteins in Swiss Prot database

**p72:** MCP_ASFB7; **A224L:** IAP_ASFB7; **p32:** P30_ASFB7; **p10:** P10_ASFB7; **p17:** P17_ASFB7; **g5R:** DIPP_ASFB7, **p12:** P12_ ASFB7; DP71L: Q65212. **eIFs from** ***Macaca mulata*** **in ENSAMB database. eIF4GI:** ENSMMUG0000010934; **eIF4E:** ENSMMUG00000011066; **eIF4A:** ENSMMUG00000009590.

## Results

### eIF4GI level remains unaltered upon ASFV infection

Very little is known about the regulation of translation in ASFV-infected cells, including the mechanisms by which ASFV shuts off host protein synthesis. ASFV infection triggers caspase-3 activation during infection [39],[40]. Since apoptosis impairs protein synthesis, leading to cleavage of eIF4GI, integrity of this initiation factor was first analyzed in ASFV-infected Vero cells. Cellular mRNA translation was inhibited at 8 hours post infection (hpi) (∼65%) (Figure 1B, lane 2) and abolished at 18 hpi, such that only viral proteins were synthesized after this time (Figure 1B, lane 3). Finally, translation of ASFV mRNAs was also inhibited at later hpi (Figure 1B, lanes 4 and 5). Interestingly, eIF4GI remained intact at 8 hpi, although cellular protein synthesis was significantly inhibited at this time (Figure 1B and C). eIF4GI cleavage products were detected from 16 hpi (Figure 1C), likely due to the effect of caspase-3 activity. In agreement with this hypothesis, procaspase-3 started to be processed to caspase-3 from 8 hpi, such that high amounts of active caspase-3 were detected after 18 and 24 hpi (Figure 1D, upper panel) [39]. However, levels of intact eIF4GI and eIF4GII were not significantly altered under these conditions (Figure 1C and D, bottom panel). To assess whether caspase-3 is responsible of eIF4GI proteolysis upon ASFV infection, eIF4GI cleavage products induced by the apoptosis inductor staurosporine (STP) in uninfected Vero cells were examined. Similar eIF4GI-derived polypeptides were observed both in ASFV-infected cells and in STP-treated Vero cells (Figure 1E, lanes 4 and 5 vs 7). In both cases, caspase-3 was detected by Western blot, suggesting the induction of apoptosis (Figure 1E, bottom panel). Moreover, eIF4GI cleavage products in ASFV-infected cells were prevented by addition of caspase inhibitors Q-VD-Oph or Z-VAD, to the culture medium (Figure 1E, lane 8 and 9). Therefore, steady-state levels of intact eIF4GI and eIF4GII were not significantly diminished throughout the infection course, suggesting that the pool of intact eIF4G is enough to support cap-dependent translation in ASFV infected cells.

### ASFV hinders eIF2α phosphorylation and induces the activation of cap-dependent translation factors

**Figure 2:**
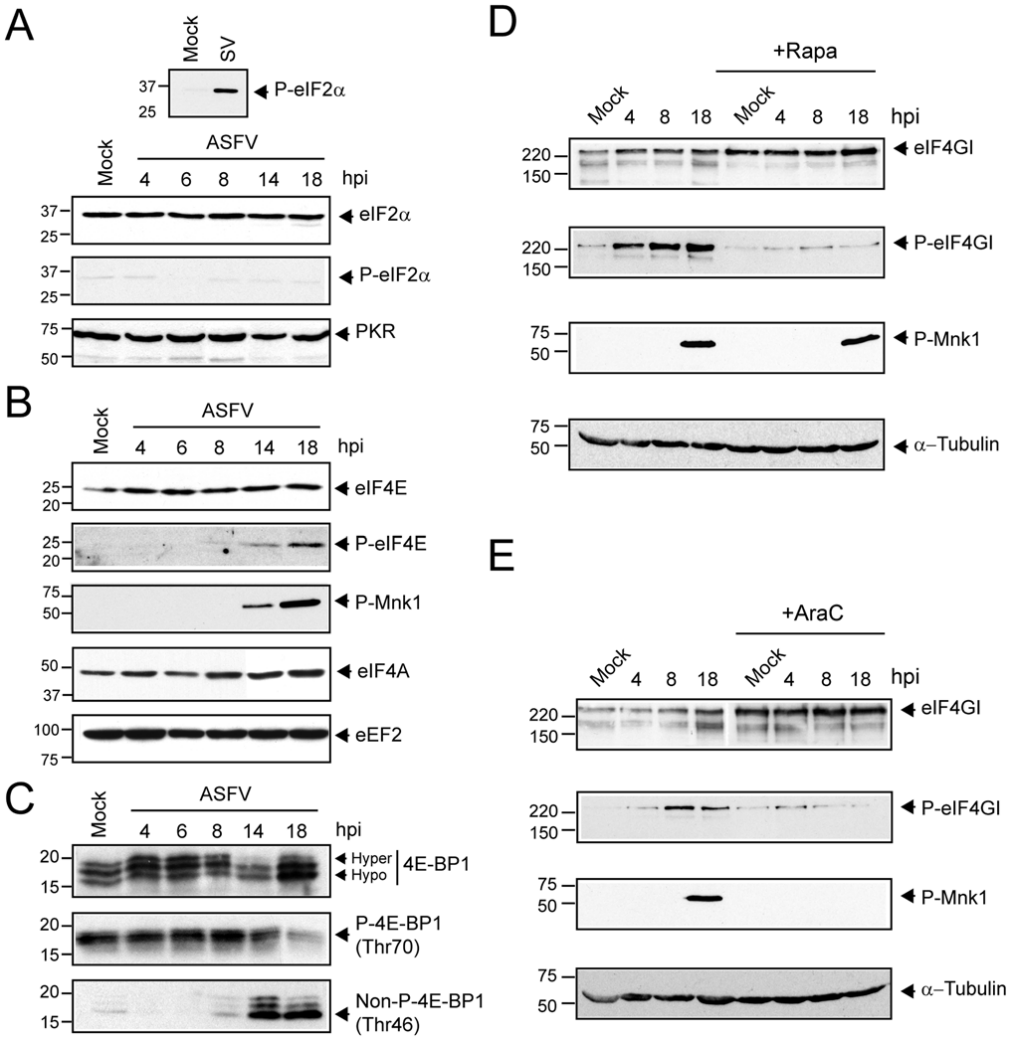
Effect of ASFV infection on total level and phosphorylation status of eIF2α, eIF4E, eIF4GI and 4E-BP1. A) Steady-state levels of eIF2α, phospho-eIF2α and PKR. At the indicated times after ASFV infection, Vero cells (MOI = 5 pfu/cell) were solubilised in sample buffer and equivalent amounts of protein were analyzed by Western blot with specific antisera. B) Phosphorylation of Mnk-1 and eIF4E is stimulated in ASFV-infected Vero cells. Vero cells were either mock-infected (Mock) or infected with ASFV (MOI = 5 pfu/cell). At the indicated times (hpi), total protein was isolated, and equivalent amounts were fractionated by SDS-PAGE, and analyzed by immunoblotting using antisera recognizing phospho-eIF4E (P-eIF4E), total eIF4E, phospho Mnk-1 (P-Mnk-1), eIF4A and eEF2. C) The phosphorylation status of 4E-BP1 was analyzed using antibodies against total 4E-BP1 (upper panel), phopho-4E-BP1 (Thr70) (middle panel) and non-phospho-4E-BP1 (Thr46) (bottom panel). D) ASFV infection increases level of phosphorylated eIF4G through mTOR activation. Vero cells were non-treated or pretreated for 12 h with rapamycin 250 nM and then infected with 5 pfu/cell of ASFV. Cells were then lysed with buffer sample at 4, 8 and 18 hpi. Amounts of phospho-eIF4GI and total eIF4GI were analyzed by Western blot (upper panels). The phosphorylation status of Mnk-1 in the absence or presence of rapamycin was analyzed by using specific antiserum anti-phospho-Mnk-1 (middle-bottom panel). α-Tubulin was detected as a load control (bottom panel). E) The ASFV-induced phosphorylation of eIF4G and Mnk-1 requires late events of the viral cycle. ASFV-infected Vero cells were treated with AraC (40 µg/ml) throughout the infection course. Cells were then lysed and 30 µg of protein were subjected to electrophoresis and analyzed by Western blot with specific antisera against eIF4GI, phospho-eIF4GI (upper panels) and phospho-Mnk-1 (middle-bottom panel). α-Tubulin was detected with a specific antibody as a load control (bottom panel).

A number of cellular protein kinases phosphorylate eIF2α [20],[21]. RNA-dependent protein kinase (PKR) and PKR-like endoplasmic reticulum kinase (PERK) are activated during many viral infections by the generation of viral dsRNA or by the accumulation of viral proteins in the endoplasmic reticulum (ER), respectively [41],[42]. Recently, it has been proposed that GCN2 phosphorylates eIF2α upon binding to specific viral RNA sequences [43]. In most cases, phosphorylation of eIF2α blocks viral protein synthesis, hampering virus spread [3]. Phosphorylation of eIF2α was examined in extracts from ASFV-infected cells by Western blot analysis using a specific phospho-eIF2α antibody. As a positive control of eIF2α phosphorylation, Vero cells were infected with Sindbis virus (SV) [44]. Infection with SV led to a potent phosphorylation of eIF2α compared to mock-infected cells (Figure 2A, upper panel), whereas phosphorylated eIF2α was undetectable in ASFV-infected cells throughout infection (Figure 2A, bottom panel). In addition, eIF2α levels were similar in each lane (Figure 2A, bottom panel), reflecting that eIF2α remains unphosphorylated in ASFV-infected cells despite the fact that PKR levels remained unaltered (Figure 2A, bottom panel). Thus, these data indicate that ASFV prevents eIF2α phosphorylation in infected cells, although the molecular mechanism involved is still unknown.

Many RNA and DNA viruses alter the activity of eIF4F by modifying the phosphorylation status of some of its components [2],[16]. To determine whether eIF4E is modified on ASFV infection, samples were taken at 4, 6, 8, 14 and 18 hpi and phosphorylation of eIF4E was analyzed by Western blot. Notably, eIF4E phosphorylation increased after ASFV infection of Vero cells (Figure 2B). Indeed, maximal eIF4E phosphorylation was achieved at 14–18 hpi, correlating with robust synthesis of viral protein (Figure 2B, lanes 5 and 6 vs Figure 1B, lane 3), while levels of total eIF4E or eIF4A were similar in each case (Figure 2B). Mnk-1 is responsible for eIF4E phosphorylation at Ser209. This kinase is activated by phosphorylation by p38 or Erk [17]. Interestingly, ASFV infection led to a potent phosphorylation of Mnk-1 at 14 and 18 hpi, which correlated with a significant increase of eIF4E phosphorylation (Figure 2B). It is well established that 4E-BPs act as inhibitors of cap-dependent translation, since they compete with eIF4G for eIF4E binding. The inhibitory activity of 4E-BPs is modulated by phosphorylation, mediated by mTOR kinase [9],[45]. To detect the phosphorylation status of 4E-BP1 in ASFV infected cells, antibodies raised against total 4E-BP1, phosphorylated 4E-BP1 at Thr70 or non-phosphorylated 4E-BP1 at Thr46 were used in Western blotting assays. The antibody against total 4E-BP1 pool identified three polypeptides with different electrophoretic mobility, corresponding to different levels of 4E-BP1 phosphorylation [16]. In contrast to that observed in VV- and HSV-1-infected cells, 4E-BP1 was hyperphosphorylated at early times post infection, but it was progressively dephosphorylated from 14 hpi (Figure 2C, upper panel). These results were further reinforced by the observations obtained with the specific antibodies that recognize phosphorylated and non-phosphorylated forms of 4E-BP1. Thus, phosphorylation at Thr70 of 4E-BP1 was shown to be significant at early times post infection, decreasing gradually from 14 hpi (Figure 2C, middle panel). Consistent with these findings, the level of non-phosphorylated 4E-BP1 dropped at 4 hpi, but increased at 14 and 18 hpi (Figure 2C, bottom panel). On the other hand, we were unable to detect a decrease on 4E-BP1 levels throughout the infection course (Figure 2C), in contrast to the results described for VV-infected cells [16]. Therefore, we can conclude that 4E-BP1 is phosphorylated at early times upon ASFV infection, whereas it is progressively dephosphorylated from 14 hpi.

eIF4GI is phosphorylated at Ser1108 in response to serum stimuli and upon the infection of many DNA viruses [8], [46]–[48]. To examine whether eIF4GI is phosphorylated after ASFV infection, a Western blot using specific phospho-eIF4GI antibody was carried out. Notably, eIF4GI was potently phosphorylated at Ser1108 in ASFV-infected cells at 4, 8 and 18 hpi, while total eIF4GI remained unaffected (Figure 2D). The phosphorylation of eIF4GI coincides with the highest ratio of ASFV protein synthesis observed from 8 to 18 hpi (Figure 1B, lanes 2 and 3). In order to test the participation of mTOR in the phosphorylation of eIF4GI, Vero cells were pre-incubated for 12 h with 250 nM rapamycin and then infected with ASFV (MOI 5 pfu/cell) in presence of the compound. Under these conditions, eIF4GI was kept unphosphorylated in both mock and ASFV-infected cells throughout the infection course (Figure 2D). These data support the idea that ASFV requires mTOR activity to induce the phosphorylation of eIF4GI. It is noteworthy that Mnk-1, which is not a substrate for mTOR [17], was phosphorylated in ASFV-infected cells despite rapamycin incubation (Figure 2D). Cytosine arabinoside (AraC) blocks the late phase of gene expression in ASFV-infected cells [39]. To elucidate whether ASFV late proteins are required to provoke eIF4GI and Mnk-1 phosphorylation, Vero cells were infected with ASFV and immediately treated with AraC. In the presence of AraC, eIF4GI as well as Mnk-1 were not phosphorylated (Figure 2E). These findings reveal that both eIF4GI and Mnk-1 phosphorylation requires the expression of ASFV late genes.

**Figure 3:**
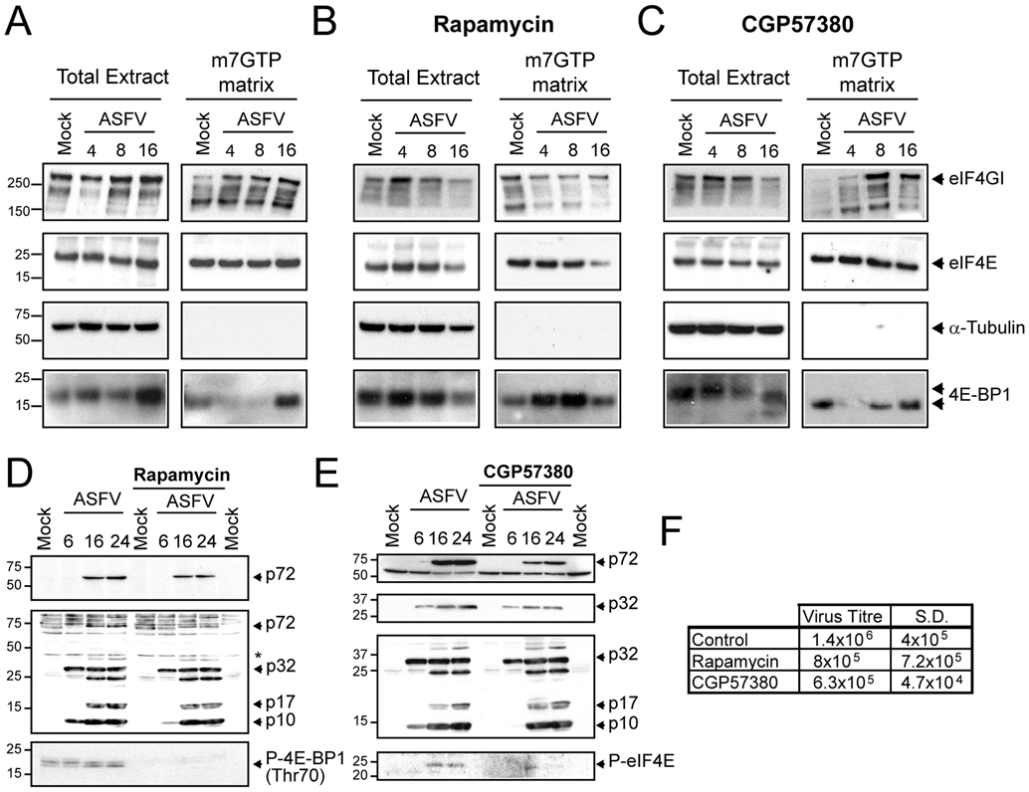
Analysis of cap-binding complex during ASFV-infection. A) Interaction of eIF4GI with eIF4E is increased upon ASFV infection. Vero cells were infected with 5 pfu/cell of ASFV and lysed at 4, 8 or 16 hpi with buffer A. Cell extracts were then incubated with Sepharose 4B matrix followed by Sepharose-4B-m7GTP matrix. Cap-binding complexes were eluted with Laemmli sample buffer. eIF4E, eIF4GI, α-Tubulin and 4E-BP1 were detected in total and eluted fractions by Western blotting. B and C) eIF4GI-eIF4E association is abrogated by rapamycin but not by CGP57380. Vero cells were pretreated with 250 nM rapamycin (B) or 20 µM CGP57380 (C). After 12 h cells were infected with ASFV (5 pfu/cell) in the continuous presence of the compounds. At 4, 8 and 16 hpi cells were recovered in buffer A and then incubated with Sepharose 4B matrix followed by Sepharose-4B-m7GTP matrix. eIF4GI, eIF4E, α-Tubulin and 4E-BP1 were detected in total and eluted fractions by Western blotting. D, E and F) Rapamycin and CGP57380 treatments only partially inhibit ASFV protein synthesis and virus spread. Vero cells were either pretreated or not with 250 nM rapamycin (D) or 20 µM CGP57380 (E). After 12 h cells were infected with ASFV (5 pfu/cell) in the continuous presence of the compounds. At 6, 16 and 24 hpi cells were recovered and viral proteins were analyzed with p72 (D and E, upper panel) and p32 (E, upper-middle panel) antibodies or an antisera that recognize most of the ASFV structural proteins (D and E middle-bottom panel). Phosphorylation of 4E-BP1 or eIF4E was analyzed with phosphospecific antibodies against Thr70 (D, bottom panel) and Ser209 (E, bottom panel), respectively. F) After 48 h of ASFV infection, supernatants from rapamycin or CGP57380 treated or untreated cells were recovered. Lytic viruses were titrated in Vero monolayers and plotted in the table. S.D., standard deviations.

eIF4E is sequestered by 4E-BPs when cap-dependent translation is inhibited. However, several stimuli elicit the disruption of this complex leading to the interaction of eIF4G and eIF4E, in turn activating cap-dependent translation [9]. Since eIF4E and eIF4GI phosphorylation is altered in ASFV-infected cells, the ability of eIF4GI to bind eIF4E was assayed. To this end, extracts derived from mock or ASFV-infected cells (MOI = 5 pfu/cell) were incubated with a sepharose-4B-m7GTP matrix or sepharose-4B as a control. After pull down, the amount of eIF4E and eIF4GI were examined by Western blot. Similar quantities of eIF4E, eIF4GI and α-Tubulin were detected in total fractions from mock and ASFV-infected cells. Furthermore, the amount of eIF4E associated with sepharose-4B m7GTP matrix was similar in extracts from mock or ASFV-infected cells (Figure 3A). As expected, eIF4E was not detected in control sepharose-4B fractions (data not shown). Interestingly, eIF4GI increased in sepharose-4B-m7GTP matrix fractions from 4 to 16 hpi, compared with mock cells (Figure 3A). This result indicates that enhancement of eIF4F assembly takes place at early times post infection. In contrast, α-tubulin was only found in the total extracts of both infected and uninfected cells (Figure 3A). 4E-BP1 disappeared from the m7GTP matrix fractions from 4 to 8 hpi, consistent with the increase of eIF4F formation (Figure 3A). It is noteworthy that although 4E-BP1 was enriched in these fractions at 16 hpi, (in agreement with the data showed above in Figure 2C), a concomitantly decrease on eIF4F assembly at this time could not be observed (Figure 3A). Further experiments beyond this work will be carry out to fully determine whether the 4E-BP1 hypophosphorylation, found at later times post infection, might be involved in the reduction of eIF4F formation and therefore possibly involved in the control of the final steps of viral protein synthesis.

Next, the role of eIF4GI, 4E-BPs and eIF4E phosphorylation in eIF4F assembly in ASFV-infected cells, was examined using specific inhibitors of either mTOR kinase (rapamycin) or Mnk1 (CGP57380). Rapamycin treatment significantly increases the association of 4E-BP1 with eIF4E and, consequently, the presence eIF4GI in m7GTP matrix fractions was not enhanced upon ASFV infection (Figure 3B upper pannel), conversely to that observed in untreated cells (Figure 3A, upper panel). Thus, we conclude that the inhibition of mTOR prevents the stimulation of eIF4F assembly detected in ASFV-infected cells. In contrast to these results, treatment with CGP57380 did not avoid the stimulation of eIF4E-eIF4GI interaction, triggered after ASFV infection (Figure 3C). In the presence of the Mnk-1 inhibitor, a lower level of 4E-BP1 was found at 4 hpi in m7GTP matrix, followed by an increase after 8 hpi (Figure 3C, bottom panel). Consequently, eIF4E-eIF4GI interaction slightly diminished at 16 hpi, therefore suggesting that the prevention of eIF4E phosphorylation observed in ASFV-infected cells, likely favour the binding of 4E-BP1 to eIF4E in a scenario in which 4E-BP1 dephosphorylation is enhancing (Figure 3C and 2C), although other hypothesis could be not discarded.

Interestingly, rapamycin, as well as CGP57380 treatment, induced a moderate inhibition of ASFV protein synthesis, since a diminution on p72, p32, p17 and p10 accumulation was achieved in treated cells, especially at early times post infection (Figure 3D and E). In addition, both compounds diminished moderately but reliably virus spread (Figure 3F).

Therefore, the increase of eIF4F assembly observed in ASFV-infected cells might enhance the synthesis of viral proteins but seems to be not essential for the progression of the infection. However, previous studies with HSV-1 revealed that the impact of Mnk-1 inhibition by CGP57380 in viral protein synthesis is inversely proportional to the multiplicity of infection used [17]. Therefore, further experiments are likely required to fully address the role of eIF4E phosphorylation in ASFV-infected cells.

### Depletion of eIF4E and eIF4GI abrogates ASFV infection

**Figure 4:**
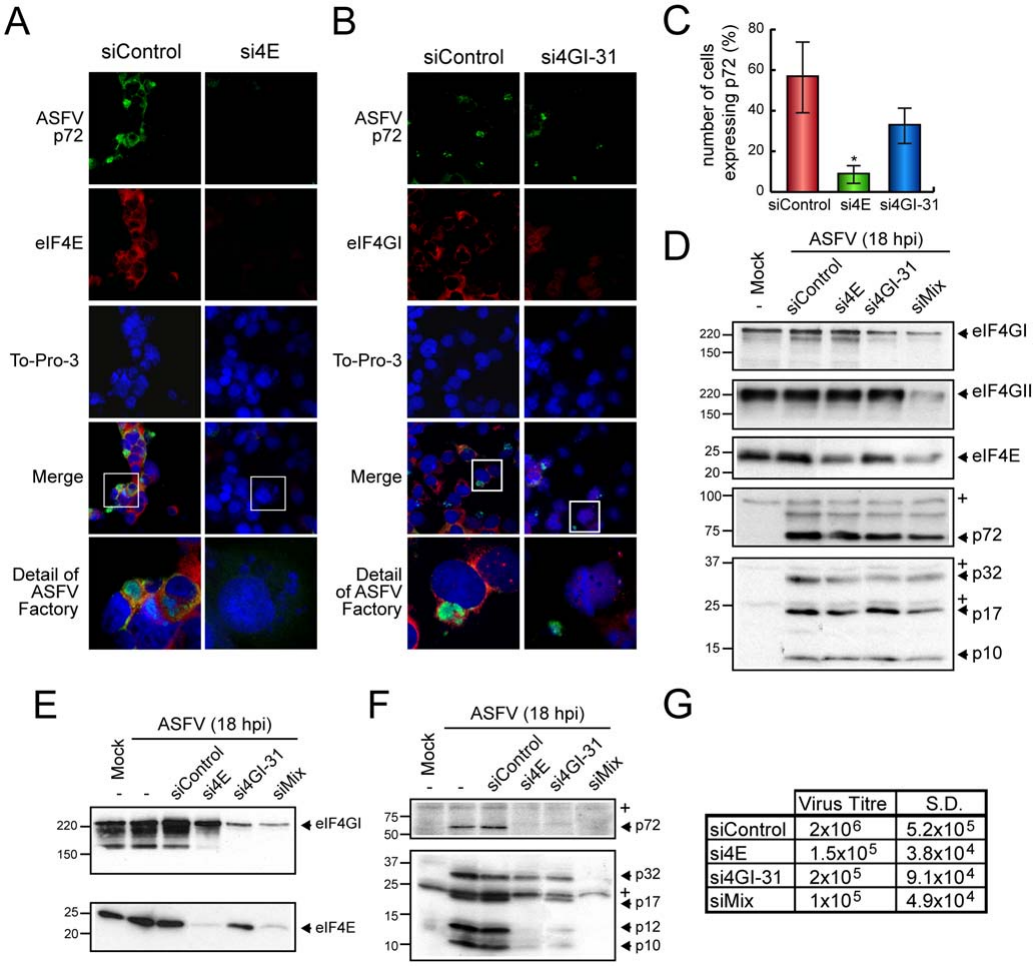
Depletion of eIF4E or/and eIF4GI blocks ASFV proteins expression in infected cells. A, B and C) COS-7 cells were transfected with siControl, si4E or si4GI-31 in 2 steps separated by 24 hours. Cells were then seeded on glass coverslips and mock infected or infected with 1 pfu/cell of ASFV. At 16 hpi, cells were permeabilised and fixed and ASFV p72 and eIF4E or eIF4GI were detected by indirect immunofluorescence. A and B) Immunofluorescence using anti-eIF4E or anti-eIF4GI, respectively, and anti-p72 in either eIF4E (A) or eIF4GI (B) silenced cells. C) Percentage of cells expressing ASFV p72 in eIF4E- and eIF4GI-silenced cells (mean±SD). * P<0.05. D) COS-7 cells were transfected with siControl, si4GI-31, si4E or a mixture of si4GI-31, si4E and si4GII-2 (siMix) as indicated. After 72 h, cells were infected with 1 pfu/cell of ASFV and samples were recovered after 18 hpi. Depletion of eIFs was examined by Western blot against eIF4GI, eIF4GII and eIF4E (upper panels). Accumulation of viral proteins was analyzed using a specific antibody against p72 (middle bottom panel) or with an antiserum that recognizes a number of structural ASFV proteins (bottom panel). E, F and G) Vero cells were transfected with siControl, si4GI-31, si4E or a mixture of si4GI-31 and si4E (siMix) as described. After 72 h, cells were infected with 1 pfu/cell of ASFV and samples were recovered after 18 hpi. Depletion of eIFs was examined by Western blot against eIF4GI and eIF4E (E). Accumulation of viral proteins was analyzed using a specific antibody against p72 (F, upper panel) or with an antiserum that recognizes a number of structural ASFV proteins (F, bottom panel). G) In parallel, supernatants from transfected cells were recovered at 48 hpi and titrated in Vero cells. Virus titre in each case was indicated in the table. * unspecific cellular protein detected by the antibody.

The above experiments illustrate that ASFV infection enhances eIF4F activity to maximize the synthesis of its own proteins. To examine the extent to which cap-dependent translation is essential for ASFV, eIF4E or eIF4GI were depleted in COS-7 cells using the previously described siRNAs [32],[33]. At 48 hours post transfection, eIF4E- and eIF4GI-silenced cells were infected with ASFV (MOI = 1 pfu/cell). At 16 hpi, immunofluorescence was performed using antibodies against eIF4E and eIF4GI. A specific antibody that recognizes ASFV-p72, the major capsid protein, which has been previously shown to localize in viral factories, was also employed to test both the infection progress and as a viral factory marker. To-Pro-3 was used in parallel to detect cellular nuclei and viral factories. As shown in Figure 4A and B, the amount of eIF4E or eIF4GI was significantly lower in cells transfected with si4E or si4GI-31 siRNAs, respectively, compared with control cells (Figure 4A and B). Interestingly, p72 was almost undetectable in most eIF4E-silenced cells (Figure 4A). In fact, the percentage of cells that synthesize p72 dropped to 5% in cells with eIF4E depletion, as compared to 60% of control infected cells that were positive for p72 (Figure 4C). Notably, in some of the eIF4E-silenced cells, several small structures appeared upon ASFV-infection, possibly corresponding to aberrant viral factories, which did not react with p72 antibodies (Figure 4A). In parallel, when ASFV-infected cells lacking eIF4GI were analyzed, the percentage of p72 positive cells not only decreased by about 20% (Figure 4B and C), but the size of the viral factories also clearly diminished as compared to non-silenced ASFV-infected COS-7 cells (Figure 4B). To further reinforce these findings, COS-7 cells were transfected with si4E, si4GI-31 or a mixture of si4E, si4GI-31 and si4GII-2. After a recovering period (72 h), they were infected with ASFV (1 pfu/cell). Cells were recovered in sample buffer at 18 hpi and eIFs and viral proteins were examined by Western blotting. A partial depletion (∼60–70%) of eIF4E, eIF4GI or/and eIF4GII was achieved using the different siRNAs (Figure 4D). These siRNAs did not induce the unspecific silencing of other initiation factors such as eIF3b, eIF2α, eIF4A and p97, as well as unrelated cellular proteins such as α-Tubulin, polyC tract-binding protein (PTB) or Ref-1 (EW, AC, PM and LC; unpublished data). However, a partial decrease of eIF4E was detected in both si4GI-31 transfected COS-7 and Vero cells. This effect was previously described [33]. Interestingly, expression of ASFV proteins was moderately reduced by silencing eIF4E or eIF4GI (Figure 4D). Notably, simultaneous knock down of eIF4E, eIF4GI and eIF4GII inhibited to a higher extent the synthesis of ASFV proteins (Figure 4D, lane 5). Depletion of eIF4E and eIF4GI was more effective in Vero cells, reaching a 90% of protein silencing in each case (Figure 4E). Under these conditions, ASFV proteins were very poorly detected by Western blot as compared to control cells (Figure 4F), especially when eIF4E and eIF4GI were simultaneously depleted (Figure 4E and F, lane 6). In order to establish the effect of eIF-depletion on ASFV production, the supernatants obtained at 48 hpi from transfected Vero cells were titrated on Vero monolayers, to analyze virus replication and spread. The amount of lytic virus produced was severely reduced (∼10 fold) by the depletion of eIF4E and/or eIF4GI, as compared to control cells (Figure 4G). Taken together, all these data indicate that both eIF4E and eIF4G are important host factors for ASFV protein synthesis and spread.

### Translation initiation factors are recruited within the viral factories in ASFV-infected cells

**Figure 5:**
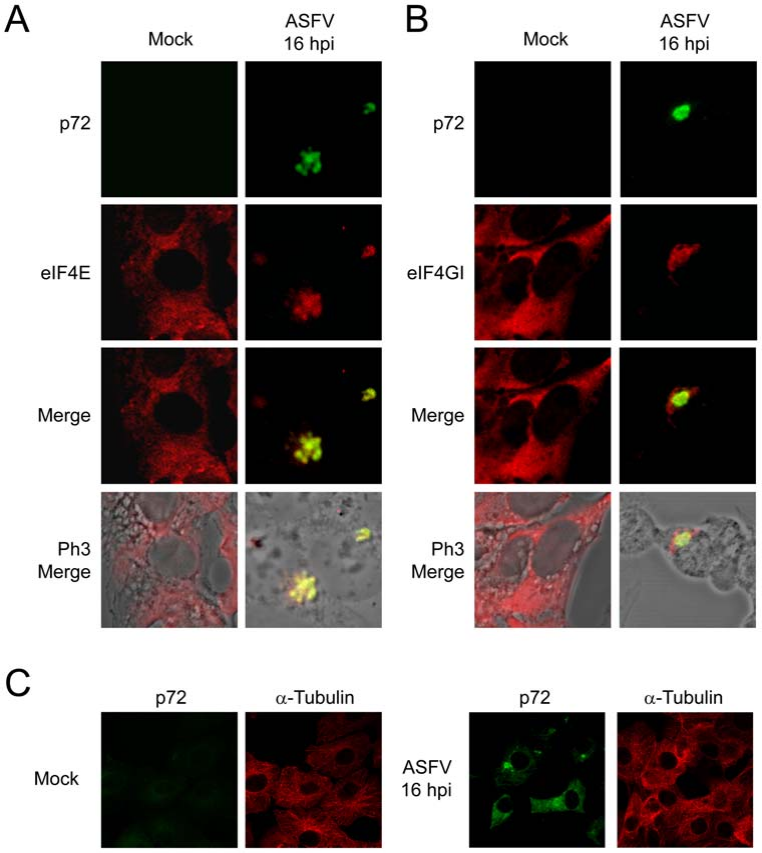
eIF4E and eIF4GI are recruited to ASFV factories in infected cells. Vero cells were seeded on glass coverslips and mock infected or infected with 5 pfu/cell of ASFV. At 16 hpi cells were permeabilized, fixed and then eIF4E (A) or eIF4GI (B) and ASFV p72 were detected by indirect immunofluorescence. Cells were visualized by confocal microscopy and the cell outline was defined by phase contrast microscopy. C) In parallel, α-Tubulin and p72 were detected in infected (right panels) and mock cells (left panels) with specific antibodies. Images were obtained under restricted conditions and processed with Huygens 3.0 software.

Two different groups have reported that eIF4GI and eIF4E are located at viral factories in VV-infected cells [16],[49]. The subcellular localization of eIF4GI and eIF4E was examined in ASFV-infected cells by immunofluorescence assays. eIF4GI and eIF4E are spread in the cytoplasm of uninfected Vero cells (Figure 5A and B), however, at 16 hpi, these initiation factors were clustered at structures akin to viral factories and partially co-localized with ASFV-p72 (Figure 5A and B). In contrast, distribution of other cellular proteins such as α-Tubulin (Figure 5C), PTB or p53 (data not shown) [39], was not significantly altered by ASFV infection. Interestingly, treatment with AraC completely prevented the redistribution of eIF4GI and eIF4E within ASFV factories, suggesting that the late viral gene expression and/or the viral factory formation are required to elicit the redistribution of eIFs (Figure S1). In addition, treatment with cycloheximide (CHX) inhibited eIF4E and eIF4GI mobilization when added at 4 hpi but not at 7 hpi, when late viral proteins were being synthesized and early viral factories can be detected (Figure S2). These data further reinforce the idea that viral factories establishment and synthesis of late viral proteins might be required for the reorganization of initiation factors in ASFV-infected cells.

**Figure 6:**
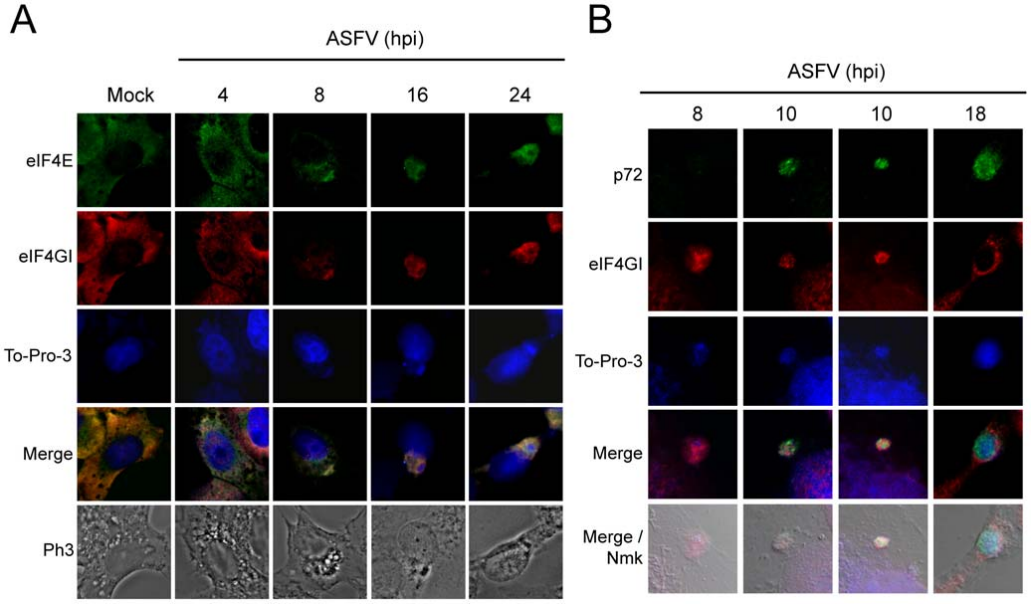
eIF4E and eIF4GI redistribution during ASFV time-infection steps. A) Vero cells were seeded on glass coverslips and mock infected or infected with 5 pfu/cell of ASFV. Cells were then permeabilized and fixed at 4, 8, 16 and 24 hpi. eIF4E and eIF4GI were detected by indirect immunofluorescence and cell nuclei and ASFV factories were stained with To-Pro-3. Cells were visualized by confocal microscopy and the cell outline was defined by phase contrast microscopy. Images were obtained under restricted conditions and processed with Huygens 3.0 software. B) Vero cells were seeded on glass coverslips and mock infected or infected with 5 pfu/cell of ASFV. Cells were then permeabilized and fixed at 8, 10 and 18 hpi. eIF4GI and p72 were detected by indirect immunofluorescence and cell nuclei and ASFV factories were stained with To-Pro-3. Cells were visualized by confocal microscopy and the cell outline was defined by nomarski microscopy.

To determine in more detail the moment when eIF4GI and eIF4E are mobilized to ASFV factories, localization of both initiation factors was carried out at 4, 8, 16 and 24 hpi. Taking into account that ASFV is a DNA virus, ToPro-3 was used to visualize ASFV replicating sites [49]. As observed above, eIF4E and eIF4GI were distributed throughout the cytoplasm of uninfected Vero cells (Figure 6A). At 8 hpi, bright ToPro-3 stained foci were detected in the cytoplasm of infected cells. Interestingly, both eIF4GI and eIF4E were found to be clustered in these foci, while the amount of these initiation factors was reduced in the rest of the cytoplasm (Figure 6A). Finally, at 16 and 24 hpi eIF4GI and eIF4E were mobilized to the periphery of viral factories, correlating with a more clear accumulation of DNA in the central area of this structure (Figure 6A). By this time after infection, a similar localization was found for other translation initiation or elongation factors such as eIF3b, eIF2α and eEF2 (Figure S3).

A further study revealed that eIF4GI is accumulated together with viral DNA at 8 hpi (Figure 6B). At 10 hpi, p72 can be detected and partially co-localized with cytoplasmic DNA, but seems to be excluded from eIF4GI sites (Figure 6B). Thus, it can be speculated that early ASFV factories might be compartmentalized in protein synthesis and morphogenesis areas. Finally, components of ASFV factories were redistributed at later times (18 hpi), showing three overlapping concentric areas: To-Pro-3 staining was more intense in the centre of the factory, while p72 accumulated at the periphery of this area and eIF4GI distributed surrounding p72 (Figure 6B).

### Localization of mRNAs in ASFV-infected cells

**Figure 7:**
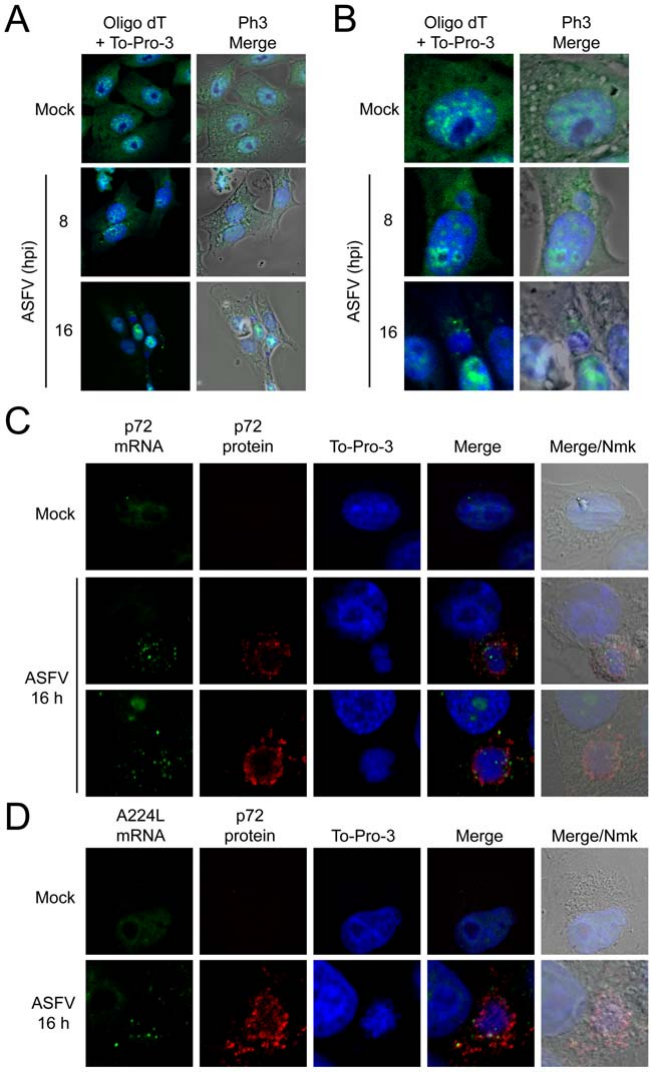
Localization of mRNAs in ASFV-infected Vero cells. Vero cells were seeded on glass coverslips and mock infected or infected with 5 pfu/cell of ASFV. At 8 and 16 hpi cells were fixed and permeabilized. Then, *in situ* hybridization with fluorescein labeled probes was carried out for each post-infection time. A) Distribution of polyadenylated mRNAs bulk by using oligo d(T) fluoresecein labelled probe. To-Pro 3 was used to stain cell nuclei and ASFV factories. B) Detail of mock infected and ASFV-infected cells, showing the polyadenylated mRNAs surrounding the viral factories. *In situ* hybridization with fluorescein labeled p72 (C) and A224L (D) probes. To-Pro 3 and p72 antibody were used to detect cell nuclei and ASFV factories. Cells were visualized by confocal microscopy and the cell outline was defined by phase contrast microscopy. Images were processed with Huygens 3.0 software.

Some DNA viruses such as HSV and VV alter cellular mRNA stability [50],[51]. To test whether ASFV affects mRNA metabolism, bulk polyadenylated mRNAs were detected in ASFV-infected cells by FISH with oligo d(T)-fluorescein. Viral replication foci were simultaneously stained with To-Pro 3. As a negative control probe oligo d(A)-fluorescein was used. In uninfected Vero cells, poly(A)-containing mRNAs localized to brightly staining foci at the nucleus, which were excluded from nucleoli (Figure 7A and B). By contrast, a diffuse distribution of cellular mRNAs was observed in the cytoplasm of Vero cells (Figure 7A and B). As expected, no fluorescence was detected in cells treated with oligo d(A) probe, indicating that oligo d(T) induces a specific fluorescent pattern (data not shown). Nuclear fluorescence increased in a time-dependent manner in ASFV-infected cells, revealing that cellular mRNA nuclear export is most likely impaired in ASFV-infected cells (Figure 7A and B). Moreover, cytoplasmic staining with oligo d(T) decreased throughout the infection course, and no mRNAs could be found in infected cells at 16 hpi (Figure 7A and B), with the exception of a polyadenylated mRNA bulk that was clustered around ASFV factories from 8 to 16 hpi. It is likely that polyadenylated mRNAs present around viral factories correspond to ASFV mRNAs, as occurs in VV infected cells [49]. These findings are also consistent with the idea that ASFV follows a strategy similar to other animal viruses to degrade cellular mRNAs.

To determine the localization of viral mRNAs in ASFV-infected cells, two fluorescein-tagged probes against p72 and A224L mRNAs were used to carry out a FISH assay. Both probes gave rise to a weak staining in the nuclei of mock infected cells, whereas no fluorescence was observed in the cytoplasm (Figure 7C and D). In agreement with oligo d(T) data, p72 and A224L probes localized in cytoplasmic granules clustered in close proximity to DNA- and p72-containing foci (Figure 7C and D). Thus, from these experiments we can conclude that late viral mRNAs are distributed at the periphery of ASFV factories, and probably are coincident with the polyadenylated mRNAs detected at these sites with oligo d(T) (Figure 7A and B vs C and D).

### Ribosomes and mitochondria are clustered at the periphery of ASFV factories

**Figure 8:**
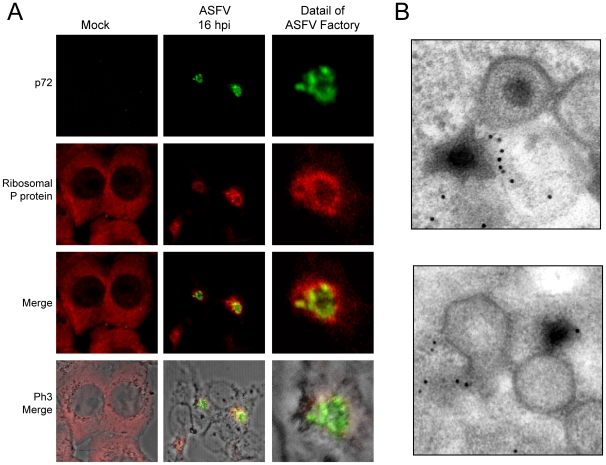
P ribosomal protein is clustered surrounding ASFV factories. A) Vero cells were seeded on glass coverslips and mock infected or infected with 5 pfu/cell of ASFV. At 16 hpi cells were permeabilized, fixed and ribosomal P protein and ASFV p72 were detected by indirect immunofluorescence by using specific antisera. Cells were visualized by confocal microscopy and the cell outline was defined by phase contrast microscopy. Images were then processed with Huygens 3.0 software. B) Electron microscopy detection of ribosomes in ASFV-assembly areas. Cells were infected with 5 pfu/cell of ASFV and processed for electron microscopy at 16 hpi. Ribosomes were detected using a specific monoclonal antibody against P ribosomal protein.

The above findings indicate that translation initiation factors are located within ASFV factories. We then hypothesized that ribosomes would be also recruited to ASFV replicative foci. To test this possibility, a specific antibody against ribosomal acidic P protein was employed, while ASFV-p72 antibody was used to detect viral factories. Ribosomal P protein was spread throughout the cytoplasm in uninfected Vero cells in a similar pattern to that of initiation factors (Figure 8A). More interestingly, ribosomal P protein partially co-localized with p72 protein in infected cells and was clustered at the periphery of ASFV factories (Figure 8A), suggesting that ribosomes are mobilized to the ASFV replicative sites. To further analyze ribosome localization, immuno-gold electron microscopy using anti-ribosomal P antibodies revealed the presence of ribosomes in ASFV factories, in the proximity of cytoplasmic regions where virus particles assemble (Figure 8B). Therefore, protein synthesis seems to be restricted to ASFV replicative foci since eIF4GI, eIF4E, eIF3b, eIF2α, eEF2 and P ribosomal protein are all within viral factories. To our knowledge, this is the first time that different components of the protein synthesizing machinery, including eIFs, eEFs, mRNAs and ribosomes, have been shown to be closely associated with viral replicative foci.

**Figure 9:**
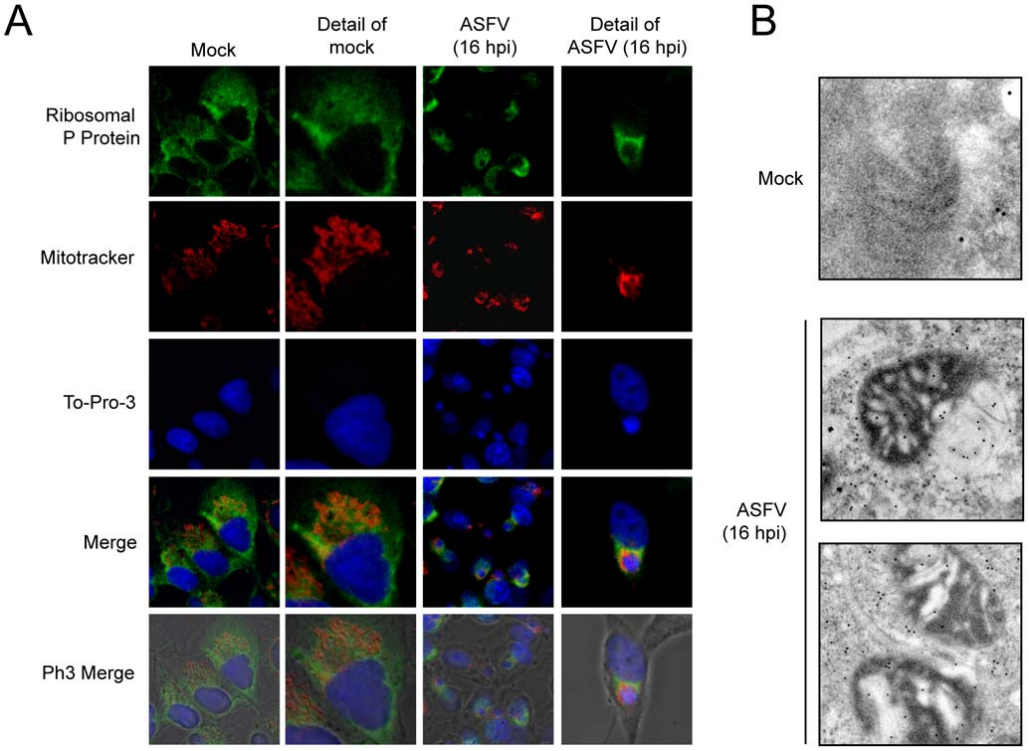
P ribosomal protein and mitochondrial network co-localize surrounding ASFV factories. A) Vero cells were seeded on glass coverslips and infected with 5 pfu/cell of ASFV. For mitochondrial staining, cells were incubated at 15 hpi with 2 µM MitoTracker red CMH2-Ros for 45 min and then permeabilized and fixed. Ribosomal P protein was detected by indirect immunofluorescence and cell nuclei and viral factories were stained with To-Pro-3. Cells were visualized by confocal microscopy and the cell outline was defined by phase contrast microscopy. Images were obtained under restricted conditions and processed with Huygens 3.0 software. B) Detection by electron microscopy of ribosomes in mitochondria-containing areas. Cells were mock infected or infected with 5 pfu/cell of ASFV and processed for electron microscopy at 16 hpi. Ribosomes were detected using a specific monoclonal antibody against P ribosomal protein.

Since viral macromolecular synthesis requires large amounts of energy in the form of ATP, we next investigated the location of mitochondria in ASFV-infected cells. In fact, previous findings from our laboratory suggested that the mitochondrial network is redistributed upon ASFV infection [52]. To examine mitochondrial distribution, the mitochondrial network was detected with MitoTracker, while ribosomes were localized using anti-P protein antibodies in ASFV-infected Vero cells at 16 hpi. The mitochondria appeared uniformly distributed in the cytoplasm of control Vero cells (Figure 9A), while accumulation of mitochondria was evident at 16 hpi at the periphery of ASFV factories, co-localizing partially with ribosomes (Figure 9A). These results indicate that the mitochondrial network and ribosomes are recruited closely to viral factories at late times of ASFV infection. These findings were reinforced by electron microscopy experiments, since we found that ribosomal P protein was enriched in the mitochondria-containing area surrounding viral factories (Figure 9B). Thus, our data provide evidence that ATP production, translation and viral replication are in close proximity to maximize the effectiveness of viral protein synthesis and DNA replication.

## Discussion

Animal viruses rely upon the host translational machinery to synthesize their own proteins since this process requires a large number of macromolecules that cannot be all encoded by viral genomes [3]. eIF4F is essential for the initiation of translation of capped cellular mRNAs, but many viruses induce, directly or indirectly, the inactivation of this complex [11]. During apoptosis eIF4G is cleaved by the cellular protease caspase-3, correlating with the inhibition of cellular protein synthesis [53]. Although ASFV has been shown to activate caspase-3 [39],[40], eIF4G cleavage does not correlate with the inhibition of cellular mRNA translation observed in infected cells. Furthermore, the level of uncleaved eIF4G present during ASFV infection did not decrease substantially, although caspase-3 derived cleavage products were detected. It can be speculated that eIF4GI could be protected from caspase-3 activity due to its location into the membranous structures, derived from endoplasmic reticulum, which constitute the ASFV factories, thus avoiding the accessibility of caspase-3 to eIF4G. Alternatively, ASFV A224L gene produces an IAP-like protein that inhibits caspase-3 activity [54]. Taking together, all these data suggest that inactivation of eIF4G by caspase-3 is not involved in ASFV-induced shut-off of host protein synthesis. In fact, we have found that eIF4G is phosphorylated at Ser1108, indicating that ASFV mRNAs most likely utilize this factor for their translation. Moreover, knock down of eIF4GI in COS-7 and Vero cells diminishes viral protein synthesis and virus spread.

eIF4E is known to be phosphorylated at Ser209 by Mnk-1, although the real function of this posttranslational modification is still controversial [9],[55]. Nevertheless, a number of viruses, such as vesicular stomatitis virus or AdV induce the dephosphorylation of eIF4E, correlating with the shut-off of cellular protein synthesis [12],[13]. In contrast, HSV-1, HCMV and VV infection trigger the phosphorylation of this initiation factor, which is concomitant with an increased association between eIF4G and eIF4E [16],[17],[19]. Mnk-1 activity plays a central role in this process, since inhibition of this protein with CGP57380 strongly reduces HSV-1 protein synthesis and virus spread, especially when low virus multiplicities were used [17]. Furthermore, inhibition of protein kinase p38 also decreased HSV-1 production, thus indicating that this kinase phosphorylates Mnk-1 in HSV-1 infected cells [17]. In addition, Erk kinase may be able to phosphorylate Mnk-1 in VV-infected cells [16]. In contrast, AdV 100K polypeptide interacts with eIF4G, thereby overlapping the Mnk-1-binding site and displacing Mnk-1 from eIF4G [13]. Mnk-1 could therefore be a key target for translational control by DNA-viruses. We show here that Mnk-1 is strongly phosphorylated at the late phase of ASFV infection, correlating with the hyperphosphorylation of eIF4E at Ser209. The inhibition of Mnk1 by CGP57380 and the subsequent dephosphorylation of eIF4E induce the increase of the association of 4E-BP1 with eIF4E from 8 hpi. This might be the cause of the reduction of eIF4GI binding to m7GTP matrix observed mainly at 16 hpi. Prevention of eIF4E phosphorylation using the Mnk-1 inhibitor CGP57380, inhibits ASFV protein synthesis and virus production. Thus, we are tempting to speculate that Mnk1 inhibition probably affects to events on the virus lifecycle or to viral factors regulating 4E-BP, rather than to a general mechanism linking inhibition of eIF4E phosphorylation with 4E-BP binding. Thus, Mnk-1 seems also to play an important role in ASFV infection, although further experiments might be carried out to further address this point. On the other hand, eIF4GI becomes hyperphosphorylated at Ser1108 early after ASFV infection (4 hpi), correlating with a significant increase on eIF4E and eIF4GI interaction. It is noteworthy that 4E-BP1 is hyperphosphorylated early after ASFV infection, concomitantly with this strong enhancement on eIF4F assembly. However, 4E-BP1 switches to a hypophosphorylated status after 14 hpi. This event might be explained as a viral mechanism to stop viral protein synthesis, when accumulation of structural proteins is sufficient to ensure viral progeny formation. Interestingly, both phosphorylation of eIF4GI at Ser1108 as well as hyperphosphorylation of 4E-BP1, are important but not essential events for ASFV infection of Vero cells, since the inhibition of mTOR by rapamycin only affects moderately viral protein synthesis and virus spread, although eIF4F assembly is inhibited under these conditions. Nevertheless, it can be speculated that changes in the phosphorylation status of eIF4GI, eIF4E and 4E-BPs could play a more relevant role on ASFV protein synthesis during the infection of swine macrophages, the natural target of the virus, since the basal levels and activity of initiation factors have been shown to be lower in differentiated cells [56].

On the other hand, our findings support the concept that cap-dependent translation is strongly stimulated after ASFV infection. The essential role of eIF4F for ASFV protein synthesis was further addressed by depletion of eIF4E, eIF4GI or eIF4GII mRNAs with specific siRNAs. Transfection of eIF4E and eIF4G siRNAs led to a significant reduction of ASFV protein synthesis, as well as virus spread. These results indicate that ASFV infection is avoided by down regulation of these translation initiation factors. Not only that, but also, it is interesting to note that eIF4E as well as eIF4GI were redistributed in ASFV-infected cells to localize within viral replicative sites at 8 hpi, while at late times of the infection, these factors were displaced to the periphery of viral factories. These findings point to the exciting idea that ASFV activates and recruits eIF4F to areas where active viral translation takes place. Such a mechanism would decrease the availability of eIF4E and eIF4G for cellular mRNA translation and may contribute to host translation shut-off. Notably, eIF4E and eIF4GI phosphorylation and redistribution are elicited during the late phase of ASFV infection, since AraC prevents all these effects. Mobilization of eIF4E and eIF4GI requires the synthesis of ASFV late proteins and, perhaps, the assembly of viral factories, given that cycloheximide added at 4 hpi but not at 7 hpi prevents this process.

eIF2α is a key target for antiviral response since regulation of this initiation factor by phosphorylation provides a rapid and reversible mechanism to regulate cellular and viral protein synthesis [3]. In fact, PERK, GCN2 and above all PKR, phosphorylate eIF2α in response to viral replication and RE stress induced by viral infection [41]–[43]. Notably, our present findings indicate that eIF2α remains unphosphorylated upon ASFV infection, suggesting the existence of a mechanism to avoid this antiviral process. ASFV DP71L protein has been shown to bind the catalytic subunit of protein phosphatase 1 (PP1), leading to PP1 activation and, consequently, eIF2α dephosphorylation [57]. Thus, DP71L has the ability to prevent the inactivation of eIF2α by a rapid dephosphorylation of this factor. However, deletion of the long form of this gene from the genome of the virulent ASFV-Malawi LIL 20/1 isolate had no effect on virulence [57], hence suggesting that the virus might further encode genes which compensate for the loss of the DP71L gene. Therefore, it is likely that additional viral mechanisms exist that target eIF2α kinases, as reported for VV K3L protein [3].

It is noteworthy that polyadenylated mRNAs progressively disappeared from the cytoplasm as ASFV infection progressed. Cellular polyadenylated mRNAs are probably degraded upon ASFV infection, consistent with the findings reported for VV-infected cells [51]. Cellular mRNAs disappeared in parallel with the inhibition of cellular protein synthesis, suggesting that this blockade might be, at least in part, a consequence of degradation of cellular mRNAs in ASFV-infected cells. In this regard, VV encodes two decapping enzymes, known as D10 and D9, which cleave the cap structure present at the 5′ end of cellular and VV mRNAs, triggering their degradation by the host machinery [58],[59]. Both VV enzymes contain a nudix domain as in cellular decapping enzymes [60]. Apparently, these enzymes do not discriminate between cellular and viral mRNAs in cell free systems [51],[58],[59]. Since VV mRNAs are compartmentalized in viral factories, they would be not available for D10 and D9 enzymatic degradation because both proteins appear spread in the cytoplasm. Alternatively, partial degradation of VV mRNAs may be compensated by their continuous *de novo* synthesis. Notably, the ASFV g5R polypeptide also contains a nudix domain [60], rendering it a good candidate for triggering mRNA decapping and subsequent degradation in ASFV-infected cells. Further studies in this new direction will be pursued with the aim of determining the functional role of g5R during ASFV infection. Furthermore, poly(A) mRNAs have been detected at the periphery of ASFV factories. Since we show here that ASFV A224L and p72 mRNAs also localize at these specific sites, the pool of polyadenylated mRNAs should correspond to viral mRNAs. Therefore, viral protein synthesis should take place in close proximity to ASFV-replicating sites.

Mobilization of eIF4E and eIF4G to viral factories has been described for VV-infected cells [16],[49]. Our present evidence indicates that not only eIF4E and eIF4G but also other initiation and elongation factors, such as eIF3, eIF2 and eEF2 and ribosomes, are recruited to ASFV factories. Taking into account that ASFV mRNAs locate at the ASFV-replicating sites, as occurs with the components of translation machinery, it is likely that viral translation should take place at these foci, supporting the idea that replication, transcription, translation and morphogenesis occur in close proximity in cytoplasmic areas coincident with ASFV viral factories. Thus, all these processes are tightly coupled, taking place in discrete cytoplasmic areas to maximize their efficiency. Interestingly, the mitochondrial network also appears to be restricted to these areas, partially overlapping with ribosomes. This novel and interesting observation could reflect the fact that ATP synthesis is also coupled to viral replication, protein synthesis and assembly. Coupling between replication, transcription and translation has been suggested for several RNA viruses [61]–[64]. Hence, in ASFV-infected cells, the proximity of the different biosynthetic machineries might help link these three events.

In summary, ASFV stimulates cap-dependent translation to increase the initiation of viral mRNA translation by activating the eIF4F complex. In order to favour viral gene expression, the protein synthesizing machinery and mitochondria are compartmentalized within viral factories. Further understanding of the mechanisms employed by ASFV to recruit these cellular components to viral replicative foci will provide additional insight into how ASFV interferes with host translation and optimizes viral gene expression.

## Supporting Information

Figure S1 Redistribution of eIF4GI and eIF4E to the ASFV factories is blocked by AraC treatment. Vero cells were seeded on glass coverslips and mock infected or infected with 5 pfu/cell of ASFV in presence or absence of AraC (40 µg/ml). At 16 hpi cells were permeabilized, fixed and indirect immunofluorescence was carried out employing specific antibodies raised against eIF4GI (A) or eIF4E (B) and ASFV p72. Cells were visualized by confocal microscopy and the cell outline was defined by phase contrast microscopy.
(2.52 MB PDF)

Figure S2 ASFV late proteins synthesis is required for the redistribution of eIF4GI and eIF4E to viral factories. Vero cells were seeded on glass coverslips and mock infected or infected with 5 pfu/cell of ASFV. At 4 or 7 hpi, CHX (10 µg/ml), was added to the culture medium and cells were fixed and permeabilized at 8 hpi. eIF4GI (A) or eIF4E (B) and ASFV p72 were detected by indirect immunofluorescence, while cell nuclei and ASFV factories were stained with To-Pro-3. Cells were visualized by confocal microscopy and cell outline were defined by phase contrast microscopy. CHX, cycloheximide.
(5.99 MB PDF)

Figure S3 eIF3b, eIF2α and eEF2 are clustered at the periphery of ASFV factories. Vero cells were seeded on glass coverslips and mock infected or infected with 5 pfu/cell of ASFV. At 16 hpi cells were permeabilized and fixed. Translation factors eIF3b (A), eIF2α (B) or eEF2 (C) were detected simultaneously to and ASFV p72 by indirect immunofluorescence with specific antisera, while cellular nuclei and viral factories were stained with To-Pro-3. Cells were then visualized by confocal microscopy and the cell outline was defined by phase contrast microscopy. Images were obtained under restricted conditions.
(2.99 MB PDF)
