# Supplementary figures and images for: Regulation of Host Translational Machinery by African Swine Fever Virus
Source: PLoS Pathog. 2009 Aug 28;5(8):e1000562. doi: 10.1371/journal.ppat.1000562 (PMC2727446; doi:10.1371/journal.ppat.1000562)

**A**

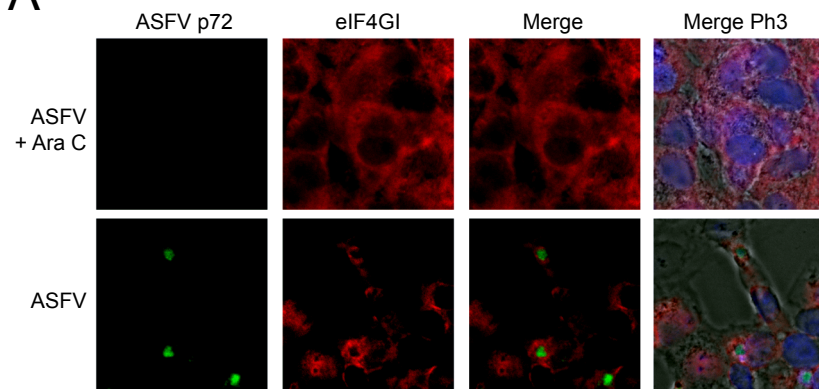

**B**

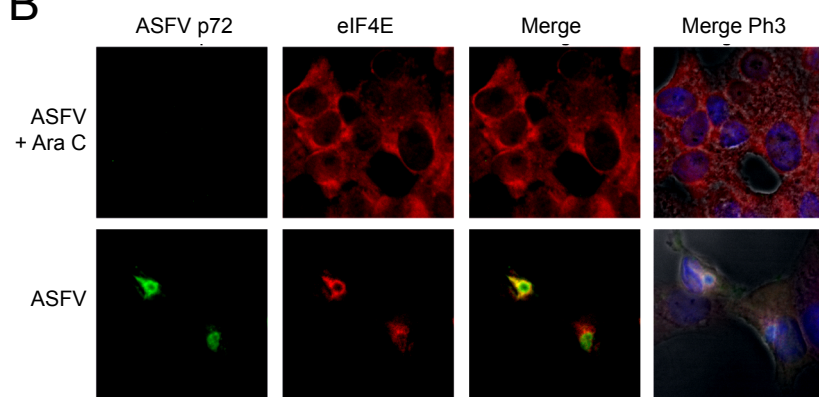

Supplement: Figure S1 — Redistribution of eIF4GI and eIF4E to the ASFV factories is blocked by AraC treatment. Vero cells were seeded on glass coverslips and mock infected or infected with 5 pfu/cell of ASFV in presence or absence of AraC (40 µg/ml). At 16 hpi cells were permeabilized, fixed and indirect immunofluorescence was carried out employing specific antibodies raised against eIF4GI (A) or eIF4E (B) and ASFV p72. Cells were visualized by confocal microscopy and the cell outline was defined by phase contrast microscopy. (2.52 MB PDF) [file ppat.1000562.s001.pdf]

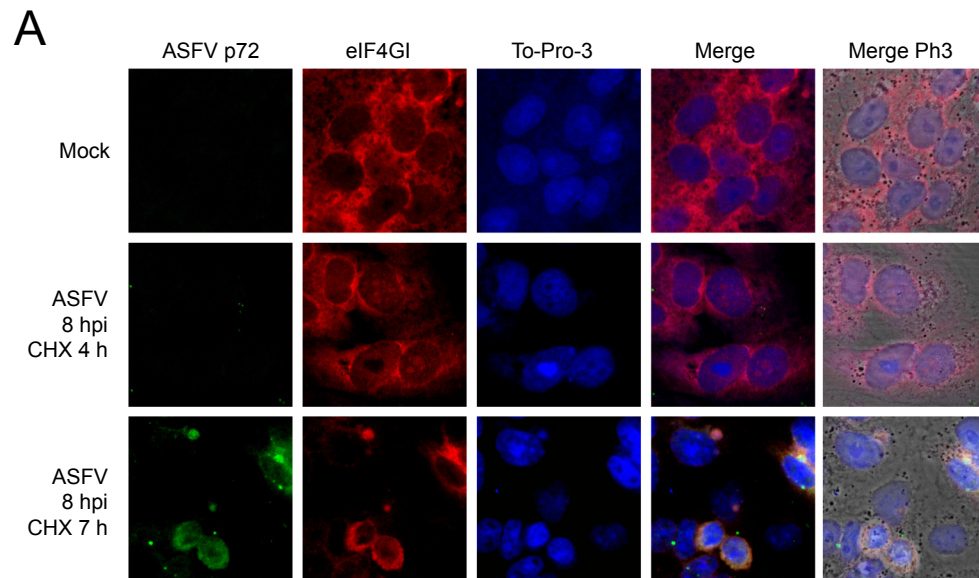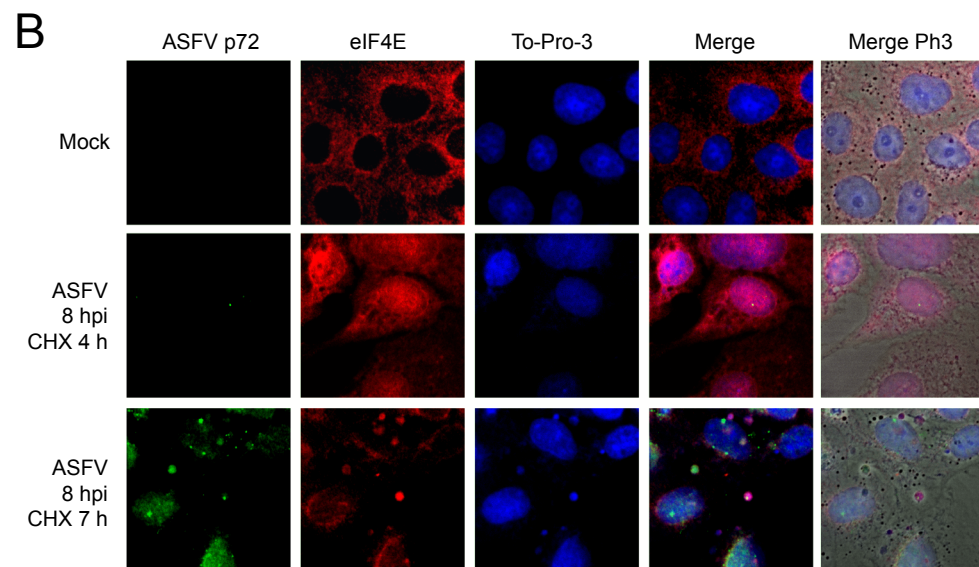

Supplement: Figure S2 — ASFV late proteins synthesis is required for the redistribution of eIF4GI and eIF4E to viral factories. Vero cells were seeded on glass coverslips and mock infected or infected with 5 pfu/cell of ASFV. At 4 or 7 hpi, CHX (10 µg/ml), was added to the culture medium and cells were fixed and permeabilized at 8 hpi. eIF4GI (A) or eIF4E (B) and ASFV p72 were detected by indirect immunofluorescence, while cell nuclei and ASFV factories were stained with To-Pro-3. Cells were visualized by confocal microscopy and cell outline were defined by phase contrast microscopy. CHX, cycloheximide. (5.99 MB PDF) [file ppat.1000562.s002.pdf]

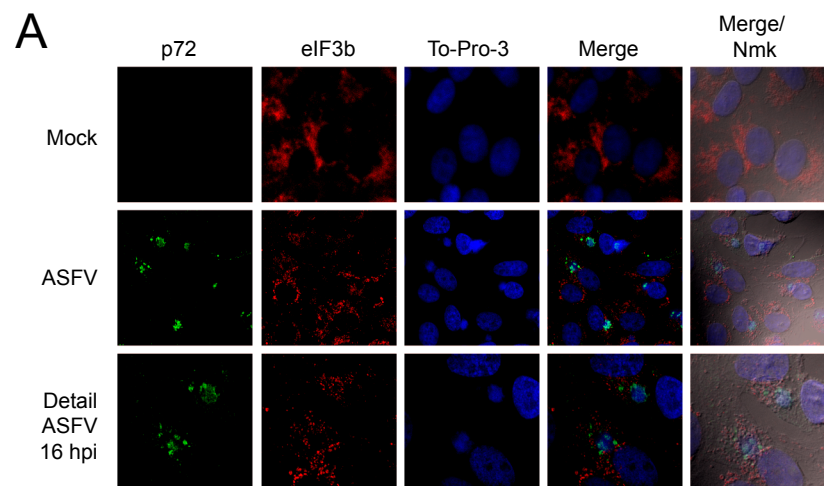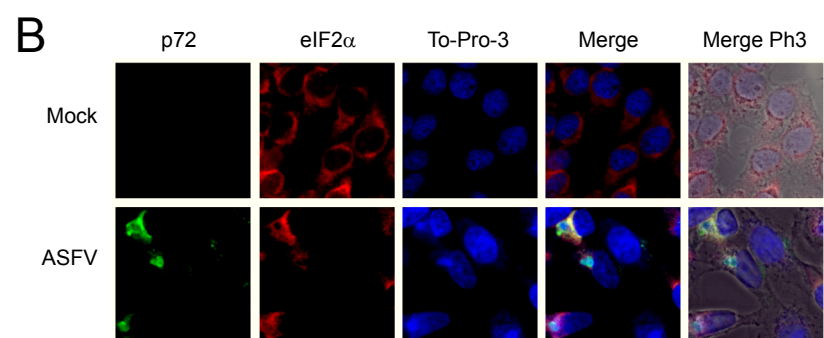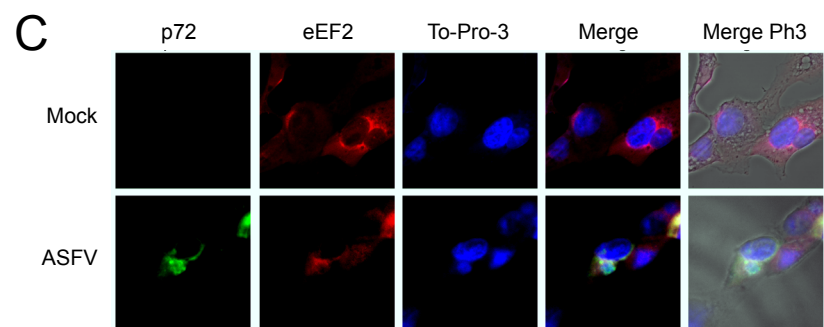

Supplement: Figure S3 — eIF3b, eIF2α and eEF2 are clustered at the periphery of ASFV factories. Vero cells were seeded on glass coverslips and mock infected or infected with 5 pfu/cell of ASFV. At 16 hpi cells were permeabilized and fixed. Translation factors eIF3b (A), eIF2α (B) or eEF2 (C) were detected simultaneously to and ASFV p72 by indirect immunofluorescence with specific antisera, while cellular nuclei and viral factories were stained with To-Pro-3. Cells were then visualized by confocal microscopy and the cell outline was defined by phase contrast microscopy. Images were obtained under restricted conditions. (2.99 MB PDF) [file ppat.1000562.s003.pdf]
